# Improving detection accuracy of heterogeneity in biological tissues through the combination of modulation-demodulation frame accumulation techniques and enhanced vgg16

**DOI:** 10.1371/journal.pone.0329009

**Published:** 2025-11-13

**Authors:** Fulong Liu, Siyuan Huang, Jie Gao, Xin Zhou, Junqi Wang

**Affiliations:** Xuzhou Medical University, School of Medical Information and Engineering, Xuzhou, Jiangsu, China; PLOS, UNITED KINGDOM OF GREAT BRITAIN AND NORTHERN IRELAND

## Abstract

Light source has obvious absorption and scattering effects during the transmission process of biological tissues, making it difficult to identify heterogeneities in multi-spectral images. This paper achieves a gradual improvement in the classification accuracy of heterogeneities on multi-spectral transmission images (MTI) through the combination of modulation-demodulation frame accumulation (M_D-FA) techniques and enhanced Visual Geometry Group 16 (VGG16) models. Firstly, experiments are designed to collect MTI of phantoms. Then, the image is preprocessed by different combinations of frame accumulation (FA) and modulation and demodulation (M_D) techniques. Finally, multi-spectral fusion pseudo-color images obtained from U-Net semantic segmentation are inputted into the original and enhanced VGG16 network models for heterogeneous classification. The experimental results show that: While both FA and M_D significantly improved the image quality individually, their combination (M_D-FA) proved superior, yielding the highest signal-to-noise ratio (SNR) and the most accurate heterogeneous classification. Compared to the original VGG16 model, the enhanced VGG16 models gradually improved the classification accuracy. Most importantly, the 3.5 Hz M_D-FA images processed by the Visual Geometry Group 16-Batch Normalization-Squeeze and Excitation-Global Average Pooling (VGG16_BN_SE_GAP) model achieved the highest classification accuracy of 97.57%, significantly outperforming results using FA or M_D alone. In summary, this paper utilizes different combinations of FA and M_D techniques to further improve the accuracy of deep learning networks on multi-spectral images heterogeneous classification, which promotes the clinical application of multi-spectral transmission imaging technology in early breast cancer detection.

## Introduction

Breast cancer has become the most common malignancy among women globally, having surpassed lung cancer, with an estimated 2.3 million new cases (11.7% of all cancer diagnoses) reported in 2020 [1]. In China, breast cancer is the leading cause of cancer-related morbidity and mortality among women, with its age-standardized rate rising by 3.3% annually over the past decade [2,3]. Regular screening is an effective method for early detection of tumors and improving patient prognosis. Early treatment of breast cancer not only preserves female breast tissue but also significantly improves the cure rate compared to patients diagnosed in the intermediate or advanced stages [4]. However, the physical structure of early breast tumors is often indistinct in medical images, making it difficult to identify and locate tumors within breast tissue in terms of position and size. Moreover, existing clinical examination techniques struggle to simultaneously meet the characteristics required for early detection of breast tumors, such as regularity, non-radiation, low cost, convenience and ease of implementation. For example, X-rays commonly used in clinical practice cannot be used to screen for early breast cancer, mainly because when X-rays irradiate human tissues, they will react with substances in the tissues, destroy the cellular structure of human tissues, and cause permanent damage to human tissues. Additionally, X-ray examinations have relatively low sensitivity for dense breast tissue in younger women [5]. Ultrasound imaging lacks standardized techniques and takes a long time, and the diagnostic ability depends on the experience and ability of technicians [6]. Computed Tomography (CT) takes a long time to scan, and only a limited number of layers can be scanned during the effective time of contrast agent, which cannot guarantee spatial resolution of images [7]. Magnetic Resonance Imaging (MRI) is slow, costly and insensitive to calcifications and cortical bone lesions, posing challenges for quantitative diagnosis [8].

In recent years, optical imaging has gradually become a research hotspot and has been widely applied in many fields. In comparison to conventional clinical imaging methods, optical imaging methods possess the following significant advantages [9]: a) The use of safe, non-ionizing radiation for non-invasive tissue detection. b) The ability to display soft tissue contrast based on optical properties. c) Potential for continuous monitoring of tissue lesions. d) High spatial resolution (with lateral resolution less than 1 micron in the visible range). Moreover, breast tissue is semi-transparent and highly transmissibility. During optical transmission imaging, tumor-associated neovascularization and elevated hemoglobin concentrations alter the optical properties (e.g., absorption and scattering coefficients) of breast tissue. These changes manifest as localized shadows in transmission images, termed heterogeneities [10]. For instance, malignant tissues exhibit higher absorption at specific wavelengths (e.g., green and near-infrared) due to dense microvasculature, whereas normal tissues show uniform transmission patterns. Our phantom experiments simulate these optical disparities using materials with controlled absorption/scattering profiles (e.g., potato blocks for low-density regions, pumpkin blocks for high-density anomalies), enabling validation of the method’s capability to detect tumor-mimicking structures. Therefore, optical transmission imaging provides a clinically non-invasive detection method for screening early-stage breast cancer.

Multi-spectral non-destructive transmission optical imaging has become a research hotspot due to its real-time, non-invasive, safe, specific and highly sensitive advantages, and has been widely applied in many fields [11,12]. However, there is relatively limited research on the application of multi-spectral transmission images (MTI) in medical field. This is mainly due to the absorption and scattering characteristics of tissues, which strictly limit the transmission depth of light sources. During the transmission process, light is absorbed by water, macromolecules (such as proteins) and pigments (such as melanin, hemoglobin) in biological tissues without being re-emitted, and the photons are lost, causing the image to become dim. The presence of these components restricts the propagation of light, making it difficult to obtain high-information images. Currently, modulation-demodulation (M_D) and frame accumulation (FA) technologies have become the most effective methods to enhance low-light signals in the process of obtaining MTI with various low-light level image detection devices. Li et al significantly improved the signal-to-noise ratio (SNR) and resolution of low-light images using FA and shaping signal techniques [13–15]. In 2019, Zhang et al reported the effectiveness of combining FA with deep learning frameworks (e.g., Faster R-CNN and SSD) for multispectral heterogeneity detection, achieving over 99.9% mean Average Precision (mAP) in simplified two-class scenarios [16]. Additionally, their earlier work introduced a joint preprocessing algorithm integrating FA and edge enhancement, which improved the peak signal-to-noise ratio (PSNR) to 57.3 dB and significantly enhanced edge contrast for transmission tissue images [17]. In 2020, our team proposed a method that combines modulation-demodulation-frame accumulation technique (MDFAT), spatial pyramid matching (SPM) model and deep learning to further improve the accuracy of heterogeneous recognition in multi-spectral images while enhancing the low-light image information content [18].

Additionally, with the advancement of machine learning and hardware capabilities, deep learning-based image object classification methods have been widely applied. Deep learning extracts high-level abstract features from images through layer-by-layer convolution and uncovers hidden properties within targets, which will facilitate the development of MTI in early breast tumor detection, bringing new possibilities to medical diagnosis [19–21]. In 2019, Ting et al developed a new algorithm called convolutional neural network (CNN) improved breast cancer classification (CNNI-BCC) using digital X-ray images [22]. Shen et al developed an end-to-end deep learning algorithm that can accurately detect breast cancer in screening mammograms while eliminating the reliance on rare lesion annotations. Moreover, this study shows that classifiers based on Visual Geometry Group 16 (VGG16) and ResNet can complement each other and preserve the full resolution of digital mammography images [23]. SanaUllah et al used the concept of transfer learning to propose a new deep learning framework for the detection and classification of breast cancer in breast cytological images. In the proposed framework, a pre-trained CNN architecture, namely GoogLeNet, Visual Geometry Group Network (VGGNet) and Residual Networks (ResNet), was used to extract features from images and feed them into the fully connected layer. Malignant and benign cells were classified using average pooling classification [24]. In 2022, Montaha et al propose a BreastNet18 model based on fine-tuned VGG16 that changes different hyperparameters and layer structures. After the ablation study of the proposed model and the selection of appropriate parameter values for the pre-processing algorithm, the accuracy of the model is superior to some of the most advanced methods available [25]. In 2023, Alexandru et al compared the efficiency of six state-of-the-art, fine-tuned deep learning models (ResNet-50, Inception-V3, Inception-ResNet-V2, MobileNet-V2, VGG-16 and DenseNet-121). These models can use transfer learning to classify breast tissue in ultrasound images into benign, malignant and normal categories. Among them, VGG-16 model obtained a good detection accuracy [26]. In 2024, Sreedhar et al proposed a hybrid CNN model combining ResNet-34, FE-VGG-16 and M-AlexNet to classify histopathological images of breast lesions in a standard dataset as benign or malignant to improve diagnostic accuracy [27]. In summary, the VGG16 network, as a deep convolutional neural network, efficiently explores the information within transmission images and identifies potential anomalous regions [28]. Moreover, the VGG16 network model demonstrates excellent performance on large-scale image datasets and can further enhance its performance through fine-tuning. Additionally, VGG16 exhibits strong generalization capabilities, making it suitable for various sizes and types of image data. Therefore, this paper selects the VGG16 network as the foundational model for heterogeneous classification and detection on MTI.

**Fig 1:**
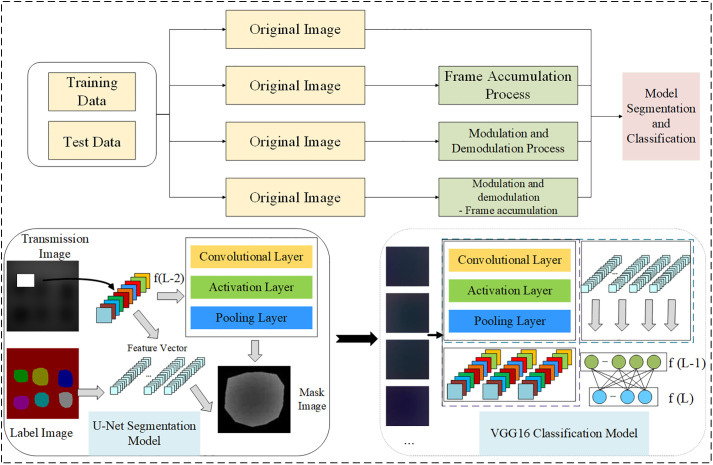
The overall classification model framework diagram.

This paper achieves a gradual improvement in the classification accuracy of heterogeneities on MTI by first demonstrating that the synergistic combination of modulation-demodulation and frame accumulation (M_D-FA) techniques provides a greater benefit than either preprocessing method alone, and then by applying these optimized images to enhanced VGG16 models. Specifically, experiments are designed to collect MTI of phantoms, and various combinations (FA, M_D, and M_D-FA) are applied for image preprocessing to rigorously compare their efficacy. The multi-spectral fusion pseudo-color images obtained from U-Net semantic segmentation are then inputted into both the original and enhanced VGG16 network models for heterogeneous classification. Both FA and M_D techniques significantly improve the image quality, and the enhanced VGG16 models gradually increase the classification accuracy of heterogeneities on transmission images compared to the original VGG16 model. In conclusion, this paper further improves the accuracy of deep learning networks in multi-spectral images heterogeneous classification by utilizing different combinations of FA and M_D techniques, building upon the enhanced image quality. The framework of heterogeneous classification detection model in biological tissues is illustrated in Fig 1.

## Related method

### U-Net network

**Fig 2:**
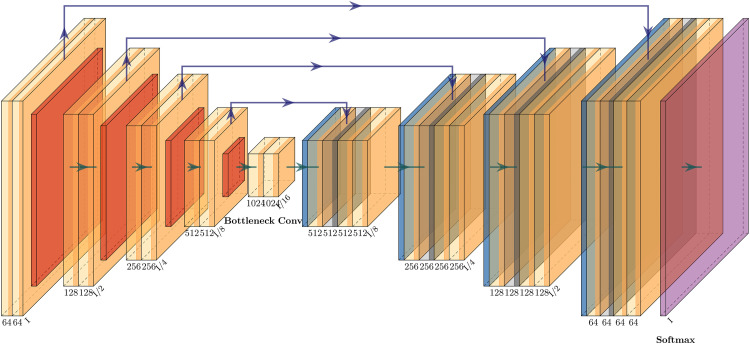
U-Net architecture model.

In medical image segmentation tasks, U-Net is one of the most successful methods. Unlike fully convolutional networks (FCN), the key differences in U-Net lie in its encoder and skip connection parts. The network structure of U-Net is fully symmetric, with similar structures on the left and right sides. U-Net fully transfers the features extracted by the decoder part to the encoder part in the skip connection part, while using concatenation operations to merge features [29]. The architecture of U-Net is shown in Fig 2. Firstly, detailed and contour information of image are obtained in the encoder part. Then, the extracted features are passed to the decoder part through the skip connection stage. Finally, the decoder part combines features from multiple scales for feature restoration. The widespread application of U-Net in the field of medical image segmentation is attributed to: (1) its ability to achieve decent results with minimal data for training. (2) the effectiveness of its network structure.

### Enhanced VGG16 network

**Fig 3:**
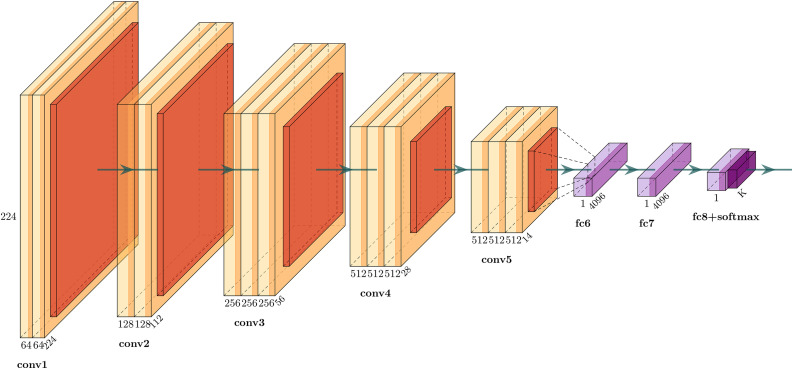
VGG16 network structure.

The VGG16 network architecture is shown in Fig 3 [30]. Its main characteristics are as follows: the convolutional layers use convolutional kernels with consistent parameters to ensure that the width and height of tensors are consistent between each convolutional layer. All pooling kernel parameters used in the pooling layer are the same, and the size is 2 × 2, stride is 2. To halve the length and width of the size after the pooling operation and reduce the number of parameters. Small convolutional kernels are stacked instead of large ones to achieve the same receptive field. For example, two 3 × 3 convolutional kernels replace a 5 × 5 convolutional kernel with the same receptive field, and three 3 × 3 convolutional kernels replace a 7 × 7 convolutional kernel with the same receptive field. Therefore, when the feature extraction effect is similar, multiple small volume kernels have fewer learning parameters than large convolutional kernels. It can also improve model performance while increasing network depth.

#### Batch Normalization (BN) layer after convolution.

The essence of the learning process in convolutional neural networks (CNNs) is to learn the data distribution. As the depth of network increases, more information can be extracted, leading to more precise classification results. However, this progress also comes with risks. With the increase in the number of convolutional layers, the training duration also becomes longer. This poses a higher risk of overfitting, resulting in poor model recognition performance and reduced network generalization ability. Moreover, the dataset is divided into multiple batches for training rather than being trained all at once during model training. When there are significant differences in the distribution of each batch of training data, the network needs to adapt to different distributions during each training iteration, thus slowing down the training process. To address these issues, the values outputted by each convolutional layer are normalized. This normalization ensures relative stability in the distribution of input data between adjacent convolutional layers, stabilizing the network training process and improving both network generalization ability and training speed [31].

#### Squeeze and Excitation (SE) attention module.

**Fig 4:**
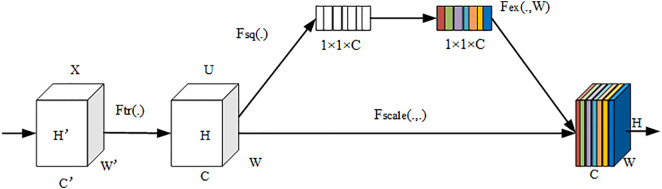
The schematic diagram of SE attention module.


The SE attention module proposed by Hu et al is one of the mainstream attention mechanisms currently [32]. It is a channel attention mechanism that calculates the weights of different feature channels in the input image through the network. This allows for the correction of feature channels, emphasizing useful information while filtering out irrelevant information, thereby enhancing the feature representation capability. Additionally, this module is lightweight and can be easily integrated into models, typically added after convolutional blocks. It only introduces a small amount of model complexity and computational overhead. The SE attention module mainly consists of two parts: Squeeze and Excitation, as illustrated in Fig 4. In the figure, $F_{tr}$ represents the convolution operation, X represents the input, U represents the output, C,W,H represent the number of channels, width and height of image, respectively. $C^{\prime },W^{\prime },H^{\prime }$ represent the number of channels, width and height of the previous image. The convolution formula is shown in Equation (1)

$$u_{c}=v_{c}*X=\sum_{s=1}^{C^{\prime }}v_{c}^{s}*X^{s}$$ (1)

Where $u_{c}$ represents the c output feature map, $v_{c}$ represents the c convolutional kernel, $v_{c}^{s}$ represents the s two-dimensional spatial kernel in the $v_{c}$ convolutional kernel, and $X^{s}$ represents the s input feature map. The generated feature maps U undergo a compression operation via a global pooling layer, denoted as $F_{sq}$, resulting in C real numbers with dimensions of 1 × 1 as shown in Equation (2). To capture correlations between channels, the compressed c real numbers undergo an activation operation denoted as $F_{ex}$, using W as the parameters to generate weights for all channels. The activation operation $F_{ex}$ is illustrated in Equation (3).


$$F_{sq}(u_{c})=\frac{\text{1}}{H\times W}\sum_{i=1}^{H}\sum_{j=1}^{W}u_{c}(i,j)$$ (2)

$$F_{ex}(z,W)=\sigma (g(z,W))=\sigma (W_{2}\delta (W_{1}z))$$ (3)

Where z represents the compressed real numbers, σ represents the sigmoid activation function, δ represents the ReLU activation function, $W_{1}\in R^{\frac{C}{r}\times C},W_{2}\in R^{C\times \frac{C}{r}}$ represents the parameter for dimension reduction. Finally, the output of the activation layer is used as a parameter to measure the importance of each channel. This parameter is added to the original features of each channel through the $F_{scale}$ operation, achieving the re-scaling of the original features weights. The formula is shown in Equation (4).


$$\overset{\sim }{X_{c}}=F_{scale}(u_{c},s_{c})=s_{c}u_{c}$$ (4)

**Fig 5:**
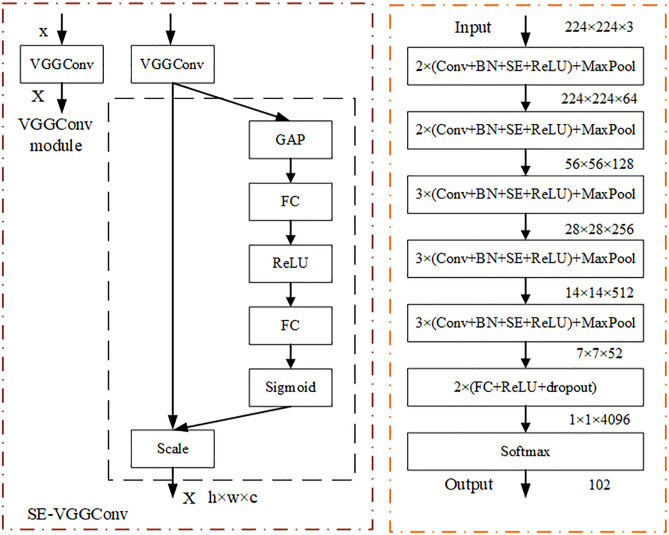
VGG16_BN_SE network architecture.

Where $\overset{\sim }{X}$ represents the re-scaled feature maps, $s_{c}$ represents the c parameter generated by the activation layer. Squeeze, as depicted in the figure $F_{sq}$, refers to the global compression feature quantity 1×1×C of the current feature map obtained by performing global pooling on the Feature Map layer. Excitation, as illustrated in the figure $F_{ex}$, refers to obtaining the weights of each channel in the feature map through two fully connected layers, and then using the weighted feature map as the input to the next layer of the network. In image classification tasks, models often experience a decrease in overall accuracy due to the omission of small and weak targets. To address this issue, this paper embeds SE attention modules after each convolutional layer in the network, as shown in Fig 5. On the one hand, this has little impact on the overall parameter quantity of model. Compared to the original model, the addition of SE attention modules only increases the parameter quantity and model size by 0.05%. On the other hand, it enhances the feature extraction capability of the network, thereby improving the model’s classification accuracy.

#### Replacing the Global Average Pooling (GAP) layer.

The VGG16 model consists of 13 convolutional layers and 3 fully connected layers, with a total parameter count of 136,349,440. The parameters of the three fully connected layers account for 89.21% of the total, resulting in excessive computational resources consumption, reduced model learning speed, increased risk of overfitting, decreased model generalization ability, and compromised real-time detection accuracy. To address the negative impact of the fully connected layers, GAP layer is introduced. By replacing the three fully connected layers in VGG16 with GAP layer, the parameter count is drastically reduced, making the model more robust and reducing the risk of overfitting. After replacing the fully connected layers with GAP layer, both the parameter count and model size decreased by 87.51%.

## Experiment

### Experimental equipment

**Fig 6:**
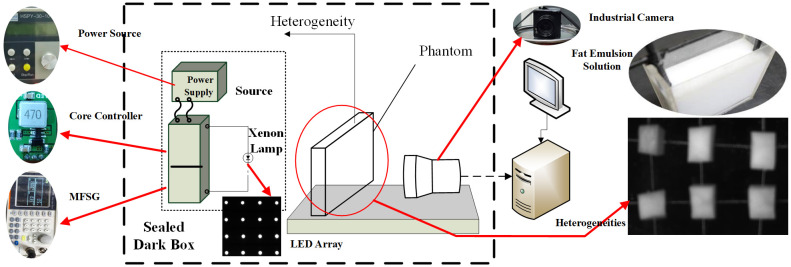
Experimental equipment diagram.

The experimental system setup is shown in Fig 6. The system mainly consists of a power supply, a modulator module, a 4 × 4 array of LED, a phantom, an industrial camera, a computer (HuiPu) and a black cloth. The power supply is a programmable direct current (DC) stabilized power supply, model hspy-600. The LED array includes wavelengths of 435nm blue light, 546nm green light, 700nm red light and 860nm near-infrared light. The illumination angle of the LED array must ensure coverage of the entire phantom area and the LED light intensity range varies sinusoidally. To simulate breast cancer scenarios based on the distinct properties of breast tissue—namely its high transmittance and tomographic distribution—the phantom is constructed using a rectangular container made of highly translucent polymethyl methacrylate (PMMA) material, chosen to replicate the tissue’s superior light transmission compared to other biological tissues. The container houses solutions of fat emulsion at three concentrations (2%, 3%, and 5%) and six heterogeneous masses (two potato blocks, two carrot blocks, and two pumpkin blocks) with controlled size variations (0.7 cm × 0.7 cm × 1 cm) to mimic tumors in breast tissue. These vegetables are selected due to their optical and structural congruence with breast heterogeneities, enabling realistic simulation of light scattering and absorption patterns. The heterogeneities are positioned at two-thirds of the phantom’s width to emulate typical tumor locations, while the experimental setup includes a light source placed directly in front of the phantom and an industrial camera positioned behind it to capture transmitted light signals. According to the concentration of the fat emulsion, the gain of industrial camera (model: JHSM120Bf, frame rate/resolution 29.4fps@1280x960, spectral response: 390–1030nm) is set to 3, 7 and 10, the exposure time is set to 5ms, 8ms and 10ms, and the sampling rate is set to 45 frames per second. The entire experiment is conducted under a black cloth.

**Fig 7:**
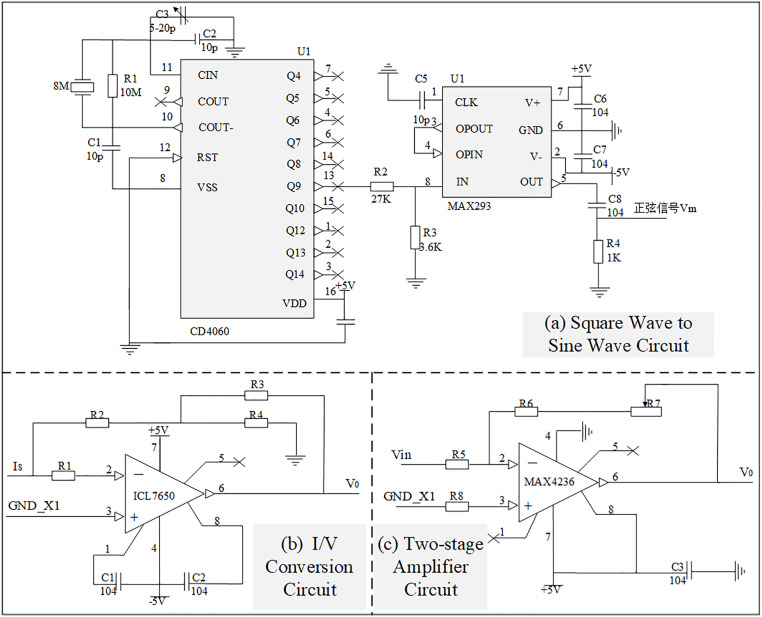
Schematic diagram of sinusoidal forming signal generating circuit. **(a)** Schematic diagram of square wave to sine wave circuit; **(b)** Schematic diagram of I/V conversion circuit; **(c)** Schematic diagram of two-stage amplifier circuit.

**Fig 8:**
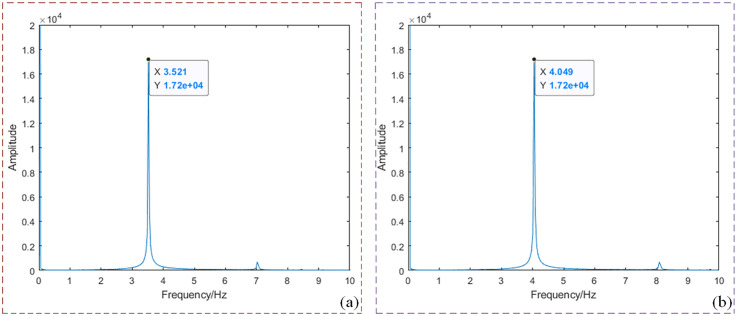
Modulation frequency diagram of shaped signal. **(a)** Frequency domain diagram of 3.5 Hz; **(b)** Frequency domain diagram of 4 Hz.

The schematic diagram of the modulator module circuit is shown in Fig 7, which utilizes a square wave to sine wave conversion circuit to generate the required 3.5 Hz/4 Hz sinusoidal signal, as illustrated in Fig 8. Fig 7a depicts the circuit schematic for square wave to sine wave conversion, where CD4060 serves as a 14-bit binary serial counter/divider, producing a high-precision square wave signal of 3.5/4 Hz at its 13th pin (Q9 pin). The precision of its output signal is fine-tuned by C3. The square wave signal, after attenuation via gain control network R2 and R3, enters the low-pass filtering circuit, retaining only the fundamental frequency signal, thereby obtaining the sine wave signal at the same frequency. The I/V conversion circuit employs the CMOS process integrated chopper-stabilized zero-drift operational amplifier ICL7650 from Maxim Integrated, as shown in Fig 7b. R1 acts as the input current-limiting protection resistor for ICL7650. The small resistors R2, R3 and R4 form a T-network, replacing the traditional use of large resistors to enhance gain stability and accuracy, and reduce noise. Additionally, for effective amplification of micro-current signals in the I/V conversion section, an inverted input type amplification circuit with a T-network is adopted, as shown in Fig 7c, producing an amplified voltage signal with a phase opposite to the input current signal.

### Image acquisition and preprocessing

**Fig 9:**
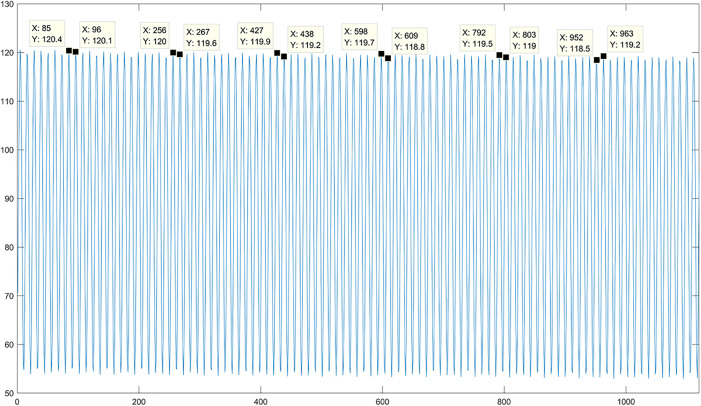
Period diagram of near infrared light sinusoidal signal.

**Fig 10:**
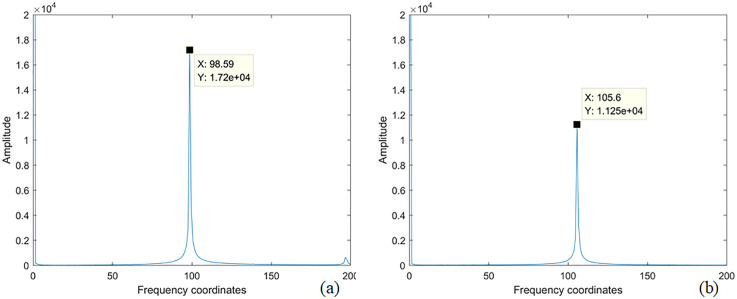
4 Hz and 3.5 Hz frequency domain coordinate diagram. **(a)** Frequency domain coordinate diagram corresponding to 4 Hz; **(b)** Frequency domain coordinate diagram corresponding to 3.5 Hz.

The original MTI are collected on the built experimental platform and the original image is processed by FA, M_D M_D-FA and SPM. The specific process is as follows:

1. Acquisition of Original Multi-Spectral Image Sequence. Four LED arrays loaded with 3.5 Hz and 4 Hz sinusoidal signal wavelengths are used to illuminate the phantom respectively, and the original MTI are obtained. Each wavelength LED array irradiates the phantom with five different concentrations (2 sets of 2% concentration fat emulsion, 2 sets of 3% concentration fat emulsion, and 1 set of 5% concentration fat emulsion). A total of 60 sets of original and modulated image data are obtained at all wavelengths, each set including 1,200 frames of images, for a total of 72,000 frames of MTI.
2. Preprocessing of Original Multi-Spectral Image Sequences. The collected MTI are processed by FA, M_D M_D-FA and respectively. a) FA processing of images. Taking a single set of multi-spectral images at near-infrared wavelength as an example, the average gray value of 1,200 low-light level images is obtained, as shown in Fig 9. It can be seen from the figure that the single cycle of the sinusoidal signal includes 12 frames of images, and each 12 frames of images is processed in order to obtain the FA image of all wavelengths, and a total of 2,000 frames FA images are obtained. b) Image M_D processing. All the modulated images obtained are subjected to fast Fourier transform (FFT) to obtain the frequency coordinates of the image loading sinusoidal signal, as shown in Fig 10. Fig 10a corresponds to the frequency domain coordinates of 4 Hz, and Fig 10b corresponds to the frequency domain coordinates of 3.5 Hz. According to the frequency domain coordinates, the multi-spectral images of all wavelengths are demodulated, and a total of 48,000 frames at different frequencies are obtained. c) Image M_D-FA processing. Similarly, the images demodulated at different frequencies are subjected to FA averaging processing based on each set of 12 consecutive frames within a single sinusoidal signal cycle. This process yields the M_D-FA images for all wavelengths, resulting in a total of 4,000 frames of images.

### Image U-Net network semantic segmentation

**Fig 11:**
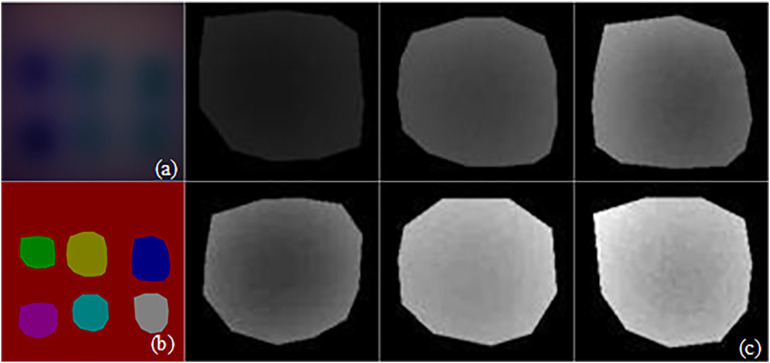
U-Net network semantic segmentation process. **(a)** Original pseudo-color image; **(b)** Semantic segmentation map of six different heterogeneities; **(c)** Mask segmentation of original image.

**Fig 12:**
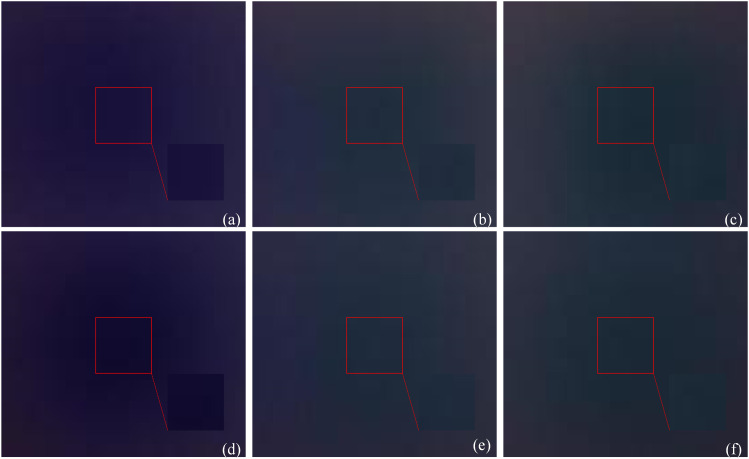
Six types of heterogeneous pseudo-color images after U-Net network segmentation. **(a)** Pseudo-color image of heterogeneity1; **(b)** Pseudo-color image of heterogeneity2; **(c)** Pseudo-color image of heterogeneity3; **(d)** Pseudo-color image of heterogeneity4; **(e)** Pseudo-color image of heterogeneity5; **(f)** Pseudo-color image of heterogeneity6. Red boxes on the main images and the corresponding zoomed-in insets highlight the representative regions of interest (ROIs), showcasing the distinct textural and contrast features discussed in the text.

**Fig 13:**
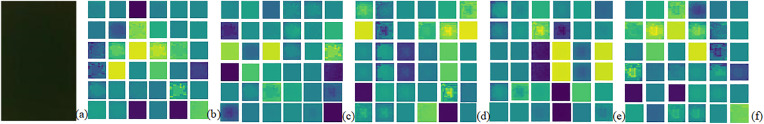
Original image and convolutional feature maps. **(a)** Pseudo-color image of heterogeneous entities; **(b)** Feature map of conv1_2; **(c)** Feature map of conv2_2; **(d)** Feature map of conv3_3; **(e)** Feature map of conv4_3; **(f)** Feature map of conv5_3.

1. MTI U-Net Semantic Segmentation. Firstly, combine the four-wavelength original MTI according to the proportions of an RGB color image to obtain a pseudo-color image, as shown in Fig 11a. Then, input the obtained pseudo-color image into the U-Net semantic segmentation network model for training, obtaining semantic segmentation annotations for the six heterogeneities in pseudo-color image, as shown in Fig 11b. Finally, acquire the original segmentation images of six heterogeneities through mask processing, as shown in Fig 11c.
2. Obtaining Pseudo-Color Images. Based on the proportional relationships of RGB primary colors in color image, recombine segmentation images to obtain original pseudo-color images, FA pseudo-color images, M_D pseudo-color images and M_D-FA pseudo-color images. The four-wavelength images are combined in the order of blue, green, red and near-infrared light, with each combination comprising every 3 wavelengths, resulting in a total of $A_{4}^{3}=24$ combinations. This yields 144,000 frames of original pseudo-color images, 12,000 frames of FA pseudo-color images, 288,000 frames of M_D pseudo-color images, and 24,000 frames of M_D-FA pseudo-color images. The obtained pseudo-color images are divided successively according to the mask region, and the 6 different heterogeneities are shown in Fig 12. While the pseudo-color images in Fig 12 may appear visually similar at first glance, a closer examination reveals that the heterogeneities exhibit distinct spectral and morphological characteristics. These differences are highlighted in the zoomed-in insets added to each panel of Fig 12. For instance, carrot blocks show stronger absorption in the red channel (700nm), resulting in darker regions in the fused images (Fig 12b and 12e, see insets). In contrast, pumpkin blocks display higher near-infrared (860nm) reflectance, visible as brighter patches (Fig 12 a and d, see insets). Potato cubes have the best light transmittance and the brightest images, with a more uniform texture (Fig 12c and 12f, see insets). These spectral differences, combined with edge/texture features extracted by the Geometry Group 16-Batch Normalization-Squeeze and Excitation-Global Average Pooling (VGG16_BN_SE_GAP) model (e.g., conv3_3 layer in Fig 13, enable accurate classification. The FA and M_D preprocessing further enhance these discriminative features by reducing noise and amplifying weak signals, as evidenced by the SNR improvements in Table 1.

**Table 1:** Comparison of SNR and PSNR for different wavelength images under different preprocessing methods.

Image/Preprocessing		Original	FA	4Hz M_D	3.5Hz M_D	4Hz M_D-FA	3.5Hz M_D-FA
Blue1	SNR1	2.7217	**---**	**---**	**---**	**---**	**---**
	SNR2	**---**	3.0073	3.4523	3.7144	3.6545	3.5015
	PSNR	**---**	86.5437	61.8956	49.8503	44.6088	42.5385
Green1	SNR1	3.4482	**---**	**---**	**---**	**---**	**---**
	SNR2	**---**	3.8539	3.8742	3.7830	3.4670	4.0724
	PSNR	**---**	49.6444	43.9290	41.2976	40.6230	43.1010
Red1	SNR1	5.3001	**---**	**---**	**---**	**---**	**---**
	SNR2	**---**	6.2385	6.1665	5.9532	5.6794	5.4931
	PSNR	**---**	47.2423	41.8101	39.6523	39.5505	41.4411
Infra1	SNR1	7.6360	**---**	**---**	**---**	**---**	**---**
	SNR2	**---**	8.5992	9.5132	9.4405	8.4726	8.0002
	PSNR	**---**	46.8916	42.9000	42.6791	45.9258	56.4111

Note: PSNR values in Table 1 are calculated relative to the original images, which serve as the reference. Consequently, PSNR is not applicable to the original (unprocessed) images themselves, as they lack a higher-fidelity ground truth for comparison.

### VGG16 heterogeneous network detection model

1. Dataset Creation. The six types of pseudo-color images combined after multi-spectral semantic segmentation are respectively made into original dataset a, FA dataset b, 4 Hz M_D dataset c, 3.5 Hz M_D dataset d, 4 Hz M_D-FA dataset e and 3.5 Hz M_D-FA dataset f. The images in dataset are divided into six categories: potato block 1, potato block 2, carrot block 1, carrot block 2, pumpkin block 1 and pumpkin block 2. Datasets a, b, c, d, e and f are randomly divided into training sets, verification sets and test sets using random functions, and the ratio is set to 6:1:3 according to the traditional partition ratio in the field of machine learning.
2. Model Training. In this study, VGG16, VGG16_BN, VGG16_BN_SE and VGG16_BN_SE_GAP networks are chosen as heterogeneous classification models. The datasets a, b, c, d, e and f are input into both the original and enhanced VGG16 networks according to the designated proportions for training. The optimal models for different datasets are established by comparing the accuracy and F-score of models. For the VGG16 network, the batch size is set to 32, Adam optimizer is utilized with an initial learning rate of 0.0001, a momentum factor of 0.9, weight decay of 0.0005 and 30 iterations with a training step size of 100. Additionally, the parameters generated by ImageNet pre-training for feature extraction are retained during training. In the SE unit module, the scaling parameter r is set to 16. To prevent overfitting, dropout of 0.5 is applied to the two fully connected layers in the 6th segment of the VGG16_SE network, while the rest of parameters are initialized using random values from a normal distribution.
3. Data Visualization. The visualization of the first 36 channel feature maps before the convolutional layers of VGG16 network is shown in Fig 13. In Fig 13, the lower layers of the network, such as conv1_2, primarily extract color and edge features from the pseudo-color images of heterogeneities. The middle layers of the network, such as conv3_3, mainly capture simple texture features of the heterogeneities. Meanwhile, the higher layers of the network, like conv5_3, predominantly extract abstract features of the heterogeneities at a finer level.

## Results and analysis

After FA and M_D processing, the quality of MTI has been significantly improved. Among them, the gray level of image is stretched, the SNR is gradually increased, and the image definition is obviously improved. The pseudo-color images with different wavelength fusion can effectively realize the classification of heterogeneities in both original and enhanced VGG16 networks.

(1) **Both FA and M_D significantly improved the quality of images.** To effectively demonstrate the changes in image quality before and after preprocessing, the SNR and PSNR are calculated before and after image processing, as shown in equations (5)-(7). The results are presented in Table 1. From Table 1, the following observations can be made: ①All wavelength images obtained from different preprocessing methods exhibit an increase in SNR compared to the original images. Higher SNR values indicate better image quality, which results in clearer visualization of the tissues. Among them, the 3.5 Hz M_D image in the blue wavelength showed the widest increase in SNR, with an increase of 36.47%. ②The PSNR values for all wavelength images under different preprocessing methods are positive. A positive PSNR value indicates a significant increase in the grayscale levels of images after preprocessing. This enhancement is beneficial for the classification of heterogeneities within the images.

In addition, we noticed that the inverse trends between SNR and PSNR in Table 1 reflect the distinct priorities of these metrics. SNR improvements (e.g., 36.47% increase for 3.5 Hz M_D in blue wavelength) confirm enhanced noise suppression, while PSNR reductions indicate deviations from the reference due to preprocessing-induced structural changes. For instance, FA reduces noise but may blur fine details, increasing MSE. Similarly, M_D-FA enhances weak signals but alters grayscale distributions, reducing fidelity to the original reference. Clinically, this trade-off is advantageous—higher SNR facilitates feature extraction for deep learning models, even at the cost of pixel-level accuracy.


$$SNR=10\mathrm{lg}[\frac{\sum_{i=1}^{m}\sum_{j=1}^{n}f{\left(i,j\right)}^{2}}{\sum_{i=1}^{m}\sum_{j=1}^{n}{\left(f(i,j)-\hat{f}(i,j)\right)}^{2}}]$$ (5)

$$MSE=\frac{\text{1}}{mn}\sum_{i=1}^{m}\sum_{j=1}^{n}{\left\|I(i,j)-K(i,j)\right\|}^{2}$$ (6)

$$PSNR=10\mathrm{lg}(\frac{MAX_{I}^{2}}{MSE})$$ (7)

Where f(i,j) represents the pixel values in image; $\hat{f}(i,j)$ represents the average value of image pixels; m×n represents the size of image; K(i,j) represents the ground truth image (Here we use the original image as the reference image.) and I(i,j) represents the processed image. Higher SNR indicates better noise suppression. Higher PSNR implies smaller pixel-level deviations from the reference. And SNR focuses on noise reduction (signal preservation vs. noise), while PSNR penalizes structural deviations from the reference (e.g., blurring or contrast shifts).

(2) **Both the original and enhanced VGG16 network models effectively achieve the classification of heterogeneities in multi-spectral images.** To comprehensively evaluate the model’s performance, this paper takes accuracy, precision, recall and F-score as evaluation indicators. True Positive (TP) represents the Positive sample predicted by the model, True Negative (TN) represents the negative sample predicted to be negative, False Positive (FP) represents the negative sample predicted to be positive, and False Negative (FN) represents a positive sample that is predicted to be negative. Recall rate and precision rate are a pair of contradictory quantities, the recall rate is relatively low when the precision rate is high. When the recall rate is relatively high, the precision rate is relatively low, so when the recall rate and the precision rate are relatively high, it means that the classification effect of this network is better. F-score is used to measure both recall and precision attributes. The definition Equations for calculating the above evaluation indicators through the confusion matrix is as follows:


$$Accuracy=\frac{TP+TN}{TP+FN+FP+TN}$$ (8)

$$\mathrm{Pr}ecision=\frac{TP}{TP+FP}$$ (9)

$$Re\mathrm{\backslash nolimits}call=\frac{TP}{TP+FN}$$ (10)

$$F1-Score=\frac{2\times \mathrm{Pr}ecision\times Re\mathrm{\backslash nolimits}call}{\mathrm{Pr}ecision+Re\mathrm{\backslash nolimits}call}$$ (11)

The enhanced VGG16 model gradually enhanced the classification accuracy of heterogeneities in multi-spectral images. Pseudo-color images fused from different wavelengths are trained separately in VGG16, VGG16_BN, VGG16_BN_SE and VGG16_BN_SE_GAP networks to achieve the classification of heterogeneities in multi-spectral images. As can be seen from Table 2: ①Compared to the original VGG16 model, the enhanced VGG16 models progressively increased the classification accuracy of heterogeneities in transmission images. The VGG16_BN_SE_GAP model exhibit the widest improvement, reaching 8.90% ± 0.75%, followed by VGG16_BN_SE and VGG16_BN models. ②Across different preprocessing methods, all models effectively improved the classification accuracy of heterogeneities compared to original images. In the VGG16_BN_SE_GAP model, the highest classification accuracy of heterogeneities in 3.5 Hz M_D-FA images is achieved, reaching 97.57% ± 0.50%. ③Among different classification models, except for the original VGG16 model achieving the highest classification accuracy for 3.5 Hz M_D images, the highest classification accuracy for heterogeneities in 3.5 Hz M_D-FA images is achieved in other enhanced VGG16 models, reaching 92.18% ± 0.65%, 95.64% ± 0.60 and 97.57% ± 0.50%, respectively. ④With the normalization of convolutional layers and the addition of GAP in the enhanced VGG16 models, the classification accuracy of heterogeneities gradually increased across different image preprocessing methods (FA, M_D, M_D-FA). ⑤In the enhanced VGG16 models, the overall average classification accuracy of 3.5 Hz image preprocessing methods is higher than that of 4 Hz image preprocessing methods.

**Table 2:** Average classification results of six heterogeneities using original and enhanced VGG16 network models.

Data set	VGG16		VGG16_BN		VGG16_BN_SE		VGG16_BN_SE_GAP	
	Accuracy%	F1-score	Accuracy%	F1-score	Accuracy%	F1-score	Accuracy%	F1-score
Original	77.01 ± 1.20	0.7679	83.11 ± 0.95	0.8307	83.93 ± 0.88	0.8402	88.45 ± 0.75	0.8822
FA	81.79 ± 1.10	0.8196	84.03 ± 0.92	0.8415	85.05 ± 0.85	0.8520	91.98 ± 0.70	0.9158
4Hz M_D	88.33 ± 0.85	0.8805	89.17 ± 0.80	0.8884	90.58 ± 0.75	0.9005	94.53 ± 0.65	0.9442
3.5 Hz M_D	91.28 ± 0.80	0.9079	91.73 ± 0.75	0.9134	93.88 ± 0.70	0.9393	94.93 ± 0.60	0.9487
4Hz M_D-FA	90.58 ± 0.75	0.9005	91.92 ± 0.70	0.9152	94.89 ± 0.65	0.9487	96.20 ± 0.55	0.9621
3.5Hz M_D-FA	88.57 ± 0.70	0.8827	92.18 ± 0.65	0.9191	95.64 ± 0.60	0.9565	97.57 ± 0.50	0.9757
Average	86.26 ± 0.90	0.8599	88.69 ± 0.80	0.8847	90.66 ± 0.74	0.9062	93.94 ± 0.63	0.9381

And the classification accuracy of heterogeneities in Table 3 reflects the model’s ability to discriminate tumor-like anomalies from normal tissue analogs. For example, H6(potato blocks) achieved 99.10% ± 1.02% accuracy in the VGG16_BN_SE_GAP model, demonstrating exceptional sensitivity to high-density, high-absorption features akin to malignant tumors. This aligns with clinical observations where malignant lesions exhibit pronounced optical heterogeneity due to irregular angiogenesis and hemoglobin accumulation. Conversely, lower accuracy for H1 (pumpkin blocks, 96.55%) suggests challenges in distinguishing subtle benign lesions from normal tissue, mirroring diagnostic difficulties in early-stage screening. The 3.5 Hz M_D-FA preprocessing method’s superior performance (97.57% ± 0.5% overall accuracy) likely stems from enhanced SNR in capturing dynamic vascular patterns, a critical factor in tumor detection.

**Table 3:** Classification results of six heterogeneities using different network models.

Different Model/Heterogeneity	H1(Accuracy%)	H2(Accuracy%)	H3(Accuracy%)	H4(Accuracy%)	H5(Accuracy%)	H6(Accuracy%)
3.5Hz M_D-VGG16	90.54 ± 1.50	91.27 ± 1.45	91.83 ± 1.24	91.09 ± 1.20	92.36 ± 1.12	90.59 ± 1.40
3.5Hz M_D-FA-VGG16_BN	91.54 ± 1.41	92.07 ± 1.15	92.36 ± 1.21	92.81 ± 1.32	93.05 ± 1.14	91.25 ± 1.20
3.5Hz M_D-FA-VGG16_BN_SE	94.52 ± 1.22	95.08 ± 1.25	95.82 ± 1.34	96.29 ± 1.14	96.74 ± 1.02	95.39 ± 1.00
3.5Hz M_D-FA- VGG16_BN_SE_GAP	96.55 ± 1.32	96.28 ± 1.35	97.02 ± 1.10	97.81 ± 1.35	98.66 ± 1.21	99.10 ± 1.02

Note: H1 = Heterogeneity1; H2 = Heterogeneity2; H3 = Heterogeneity3; H4 = Heterogeneity4; H5 = Heterogeneity5; H6 = Heterogeneity6.

## Conclusion

This study demonstrates significant translational potential for advancing early breast cancer detection through the integration of M_D-FA techniques and enhanced deep learning models. By achieving a classification accuracy of 97.57% ± 0.50% for heterogeneities in MTI, our framework provides a radiation-free, cost-effective alternative to conventional imaging modalities, directly addressing the clinical need for safer and more accessible screening tools. The experimental validation using phantoms—designed to replicate the optical properties of breast tissue—establishes a critical foundation for future clinical trials, as the controlled improvements in SNR and image clarity (e.g., 36.47% SNR increase) correlate strongly with enhanced diagnostic precision in real tissue. In preparation for these critical next steps, we are currently in the process of obtaining IRB approval and establishing collaborations with affiliated hospitals to acquire clinically annotated breast tissue images. These data will be used to validate the proposed framework in real-world scenarios, with a focus on correlating optical heterogeneities with histopathological findings. We anticipate that these efforts will facilitate the clinical translation of our method and enhance its diagnostic reliability. However, the synergistic effects among the various techniques (e.g., M_D-FA, U-Net segmentation, and VGG16 enhancements) are not yet fully explored, potentially limiting the fusion efficiency between algorithmic components. To bridge this gap and ensure robust applicability to biological tissues, future work will prioritize optimizing the integration of these techniques, validation of the model on human-derived datasets, and calibration of wavelength-specific parameters to account for tissue variability (e.g., hemoglobin absorption). Furthermore, future work must address two critical steps for clinical translation: (1) Correlation of optical heterogeneities with histopathological findings in biopsy-confirmed breast tissues; (2) Integration of clinical metadata to refine classification thresholds for benign vs. malignant discrimination. Additionally, optimizing frequency-specific modulation (e.g., 3.5 Hz vs. 4 Hz) to target tumor-specific hemodynamic patterns could further enhance diagnostic specificity.
